# Case Report: Diagnosis of Myelodysplastic Syndrome in a 72-Year-Old Female With Interstitial Lung Disease

**DOI:** 10.3389/fmed.2021.673573

**Published:** 2021-08-09

**Authors:** Nikoleta Bizymi, Georgios Pitsidianakis, Despo Ierodiakonou, Georgios Stathakis, Eirini Vasarmidi, Stavroti Hiraki, Maria Bolaki, Konstantinos Karagiannis, Michail Fanaridis, Konstantinos Liopyrakis, Leonidas Marinos, Irini Xilouri, Katerina M. Antoniou, Nikolaos Tzanakis

**Affiliations:** ^1^Department of Respiratory Medicine, University Hospital of Heraklion, Heraklion, Greece; ^2^Hemopoiesis Research Laboratory, School of Medicine, University of Crete and Department of Hematology, University Hospital of Heraklion, Heraklion, Greece; ^3^Department of Primary care and Population Health, University of Nicosia Medical School, Nicosia, Cyprus; ^4^Department of Hemopathology, Evangelismos General Hospital, Athens, Greece

**Keywords:** pulmonary infiltrates, myelodysplastic syndrome, immune deregulation, corticosteroid-therapy, mycophenolate mofetil

## Abstract

Acute fibrinous and organizing pneumonia (AFOP) is an entity that can be secondary to various conditions leading to lung injury, such as infections, malignancies, and various autoimmune conditions or idiopathic interstitial lung disease, when no obvious underlying cause is identified. Myelodysplastic syndromes (MDS), on the other hand, are a spectrum of clonal myeloid disorders, with a higher risk of acute leukemia, characterized by ineffective bone marrow (BM) hematopoiesis and, thus, peripheral blood (PB) cytopenias. Immune deregulation is thought to take part in the pathophysiology of the disease, including abnormal T and/or B cell responses, innate immunity, and cytokine expression. In the literature, there are a few case reports of patients with MDS that have presented pulmonary infiltrates and were diagnosed as having AFOP or organizing pneumonia (OP). It is rare, though, to have isolated pulmonary infiltrates without Sweet's syndrome or even the pulmonary infiltrates to precede the diagnosis and treatment of MDS, which was our case. We present a 72-year-old female developing new lung infiltrates refractory to antibiotic treatment that responded well to corticosteroids and was histologically described as having OP. The treatment was gradually successfully switched to mycophenolate mofetil (MMF). The patient was later diagnosed with MDS. This interesting case report suggests firstly that a diagnosis of AFOP or OP should alert the clinician to search for an underlying cause including MDS and *vice versa*, the use of systemic steroids should not be postponed, and, finally, that MMF can successfully be used in these patients.

## Introduction

Acute fibrinous and organizing pneumonia (AFOP) is an entity of lung injury that can be secondary to various conditions leading to lung injury, such as infections, malignancies, and various autoimmune conditions or idiopathic interstitial lung disease, when no obvious underlying cause is identified. AFOP presents with pulmonary infiltrates in lung imaging and a characteristic histological patchy pattern, with fibroblasts, foamy macrophages, and inflammatory infiltration, but with the absence of hyaline membranes, eosinophils, and fibroblastic Masson bodies. Through its healing course, it develops to organizing pneumonia (OP). This patchy characteristic makes it difficult to make a diagnosis through bronchoscopy, and also fine-needle biopsy (FNB) may be non-diagnostic. AFOP can be either acute related with high mortality and morbidity rates or subacute related with better outcomes. Patients commonly present with severe respiratory failure and need mechanical ventilation. On-time initiation of corticosteroid treatment plays a central role in the outcome, while immunosuppressive agents such as mycophenolate mofetil (MMF) have also been used (1–3). It is essential for the diagnosis to obtain negative microbiological testing, to have fluctuations of the clinical picture over time and the response to steroids, and to exclude other entities such as an underlying malignancy (4).

Myelodysplastic syndromes (MDS), on the other hand, are a spectrum of clonal myeloid disorders, with a higher risk of acute leukemia, characterized by ineffective bone marrow (BM) hematopoiesis and, thus, peripheral blood (PB) cytopenias. Immune deregulation is thought to take part in the pathophysiology of the disease, including abnormal T and/or B cell responses, innate immunity, and cytokine expression (5, 6). Autoimmune manifestations are quite common in MDS patients and may be stratified into five categories, i.e., acute systemic vasculitis or autoimmune disorder, chronic or isolated autoimmune phenomena, classical connective tissue disorders, immune-mediated hematological abnormalities, and asymptomatic serological immunological abnormalities. The pathophysiology of these phenomena, while not clear, may be attributed to this aforementioned immune deregulation (7–9).

In the literature, there are a few case reports of patients with MDS that have presented pulmonary infiltrates and were diagnosed with AFOP or OP. In some cases, this is drug related due to an agent given for the treatment of MDS or as part of the autoimmune phenomena of MDS and accompanied by other manifestations, most commonly Sweet's syndrome, i.e., fever, leukocytosis, and tender erythematous plaques. These cases are mostly treated and are responsive to steroids, and relapses are usual with their reduction. The need for mechanical ventilation, ventilatory failure, and fatality are not rare (10–15).

It is, however, rare to have isolated pulmonary infiltrates without Sweet's syndrome or even the pulmonary infiltrates to precede the diagnosis and treatment of MDS, which was seen in our case. Below, we present a case of a 72-year-old female who, prior to being diagnosed with MDS, developed new lung infiltrates in the chest imaging, refractory to antibiotic treatment and that was histologically described as OP. The patient responded well to corticosteroids and was gradually switched to MMF successfully. The positive outcome, and even without the use of non-invasive ventilation, also rare in the literature, was probably due to the early use of steroids. Furthermore, the successful switch to the sparing agent MMF, while used in AFOP related to other causes (1), was not described until now in other case reports of MDS patients with AFOP or OP.

## Case Description

A 72-year-old female, never smoker, with a medical history of chronic atrial fibrillation and heart failure under treatment with apixaban and propaphenone, visited the emergency unit of Heraklion University Hospital in June of 2019 due to fever and productive cough. The chest X-ray revealed a new infiltrate and the arterial blood gases (ABGs) at room air revealed partial pressure of oxygen (PO_2_) of 53 mmHg, partial pressure of carbon dioxide (PCO_2_) of 33 mmHg, pH 7.50, and HCO_3_ of 25 mmol/L.

Several months before, in January 2019, after an appointment with a general practitioner, thyroid, chest, and upper/lower abdomen computed tomography (CT) had been checked. Due to a small parenchymal consolidation in the right lower lung lobe, she had been referred to a pulmonologist and had been given a short course of 30 days (from April to May 2019) of methylprednisolone with a starting dose of 32 mg tapered quickly to 4 mg. In early May, due to the occurrence of fever, she had been hospitalized with sputum positive for pneumocystis pneumonia (PCP) and received a course of trimethoprim/sulfamethoxazole and prednisolone. With the reduction of prednisolone, she redisplayed fever and visited the emergency unit of the university hospital in June 2019 and was admitted to the Department of Respiratory Medicine.

On admission, she was alert with a Glasgow Coma Scale (GCS) of 15/15, body temperature of 35.8°C, pulse of 94 bpm, and blood pressure of 142/81 mmHg. She had no abnormal findings from cardiac and abdominal clinical examination, and from her chest auscultation, she had rales heard over the mid and lower right lung field. Lung ultrasound revealed consolidation in the mid and lower right lung fields and the absence of pleural effusion.

Laboratory data revealed a white blood count of 6,800/ml, hemoglobin of 9.2 g/dl, lymphocyte count of 600/ml, eosinophil count of 200/ml, and platelet count of 350,000/ml, erythrocyte sedimentation rate at 120 mm/h, C-reactive protein of 23.94 mg/dl, and international normalized ratio of 1.3; electrolytes, creatinine, the liver function tests, and urine analysis were normal.

**Figure 1:**
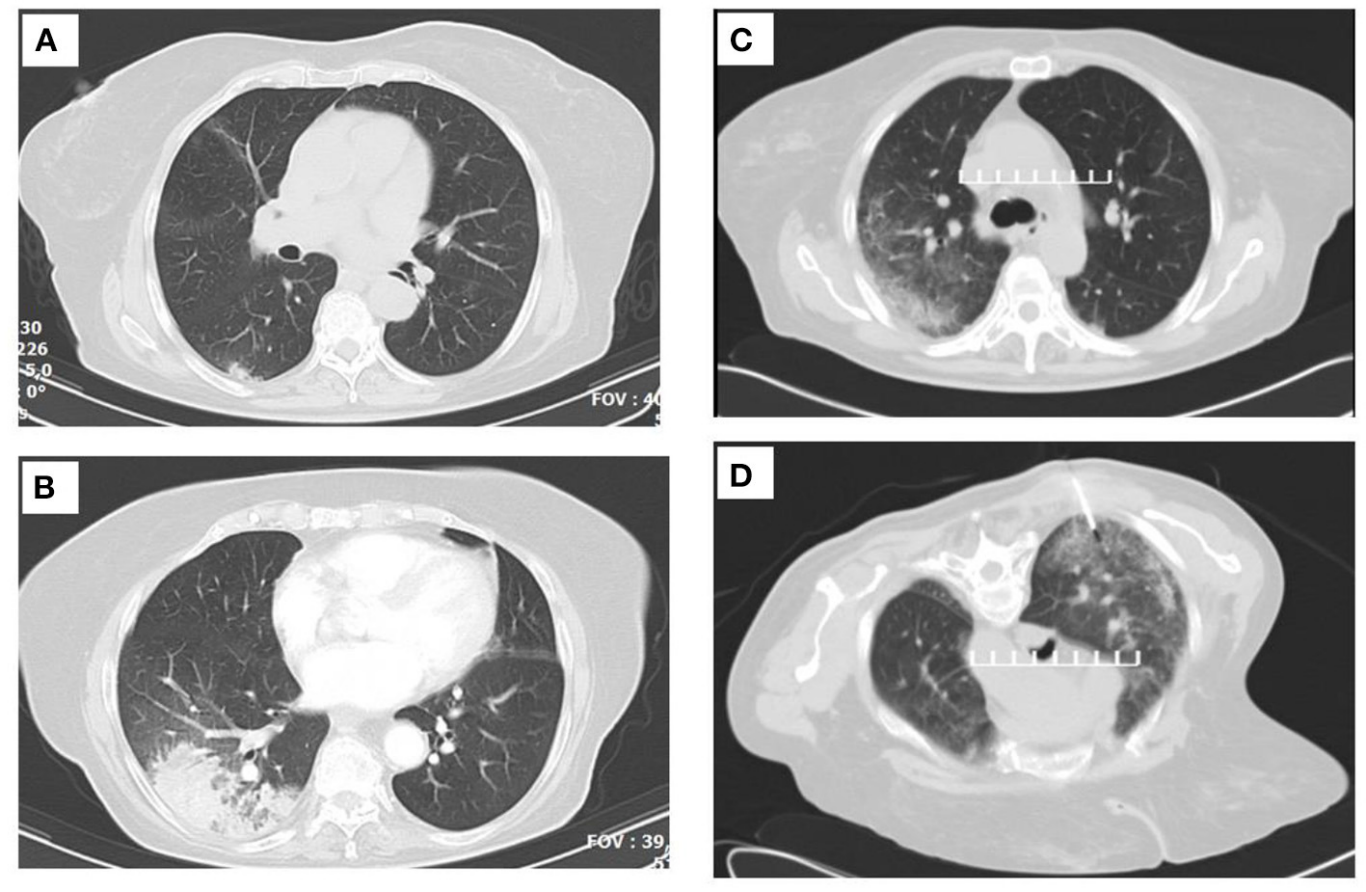
Serial thoracic computed tomographies (CTs) that demonstrate the lesion migration. **(A)** Image before corticosteroid initiation. **(B)** Image after the initial short-term corticosteroid treatment. **(C)** Image at the time of the CT-guided biopsy. **(D)** Fine-needle biopsy (FNB) cutoff point.

**Figure 2:**
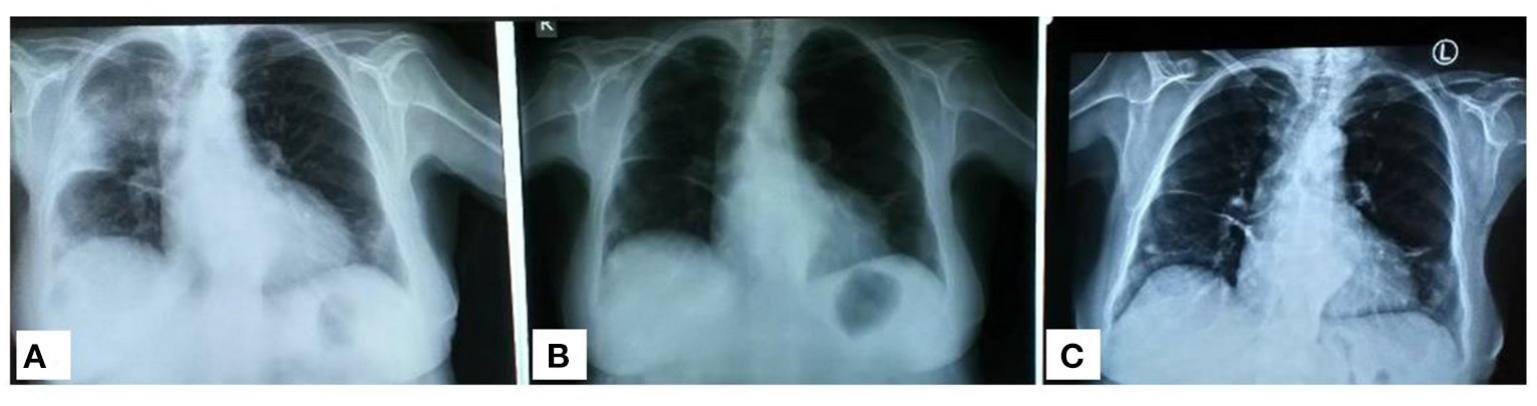
Serial thoracic X-rays that show the migration of the lesions. **(A)** Image on admission. **(B)** Picture after corticosteroid initiation as organizing pneumonia. **(C)** Picture 2 months after discharge.

The patient had negative blood and sputum cultures, negative serological test for *Cryptococcus* and *Aspergillus*, negative sputum for PCP and acid-fast bacilli, negative viral testing (including cytomegalovirus, Epstein–Barr virus, human immunodeficiency virus, etc.), and normal autoimmune testing (including serum immunoglobulins, complement segments, anti-neutrophil antibody, anti-neutrophil cytoplasmic antibody, rheumatoid factor, etc.). She had no elevated eosinophils, either in blood or in the sputum. She received oxygen therapy and broad-spectrum antibiotics (she was already on a prophylactic dose of trimethoprim/sulfamethoxazole that was continued; piperacillin-tazobactam/ciprofloxacin/linezolid were initially added and then switched to colistin intravenously and *via* inhalation/fluconazole), without improvement. Due to the persistent respiratory failure, she was not able to undergo bronchoscopy. Because of technical reasons, her appointment for CT-guided biopsy was delayed, so steroids were initiated with 40 mg intravenous methylprednisolone, and the patient rapidly improved. Figures 1, 2 include the evolution of the lung imaging of the patient.

**Figure 3:**
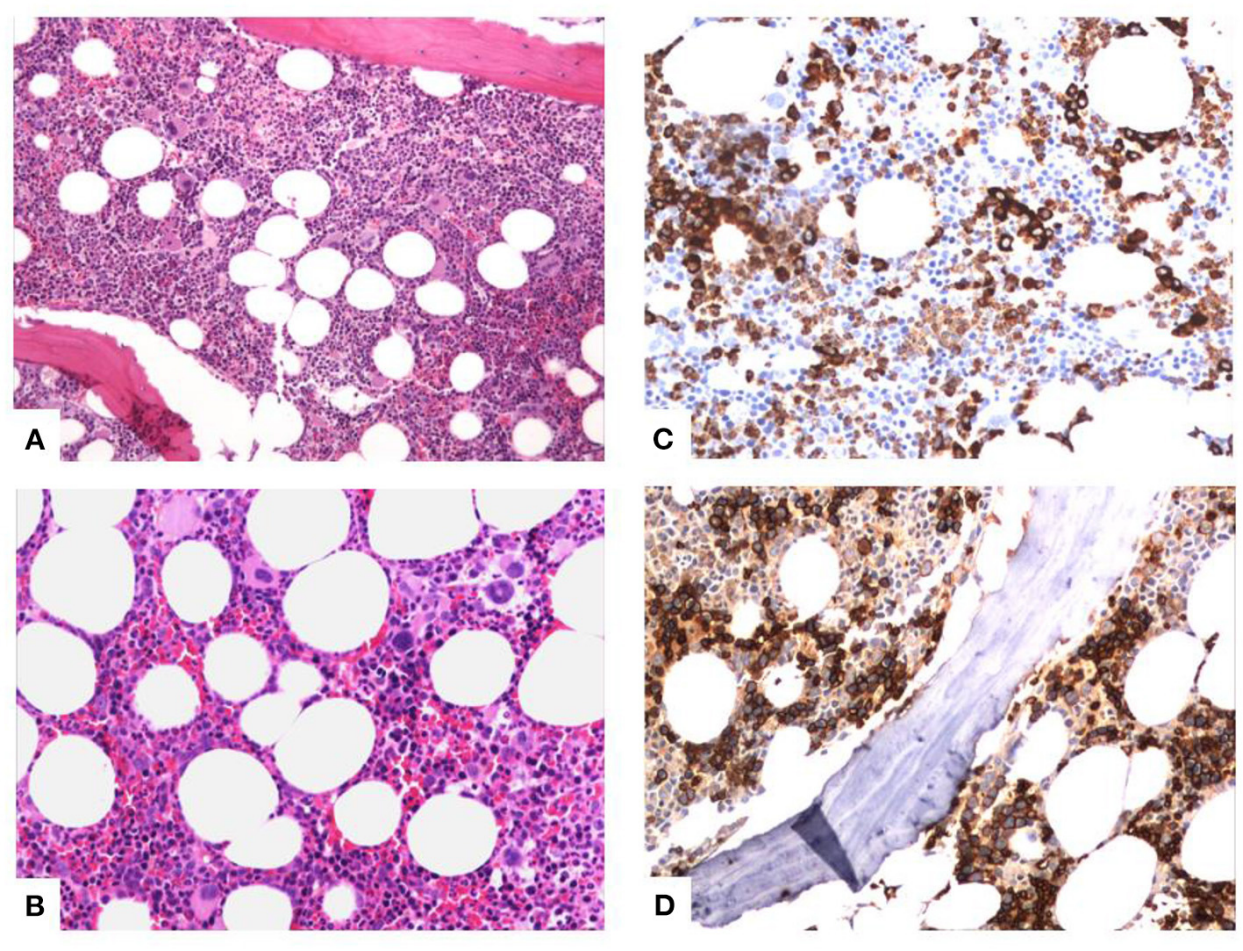
Characteristic pictures of the bone marrow biopsy. **(A)** Hypercellular bone marrow with panmyelosis (trilineage hyperplasia). **(B)** Diversity in nuclear morphology of the megakaryocytes (multi- or monolobated forms, irregular chromatin distribution). **(C)** Aggregates of immature myeloid precursors in the intertrabecular region (myeloperoxidase stain). **(D)** Abnormal localization of erythroblasts (some with megaloblastic features) on the endosteum of the trabecular bone (Glycophorin C stain).

She had a history of elevated mean corpuscular volume (MCV) in the PB with normal values of hemoglobin and hematocrit for the last years. The patient showed no deficiency of B12, folic acid, or ferritin; normal levels of thyroid hormones, reticulocytes, bilirubin, and lactic dehydrogenase (LDH); and normal urine analysis. Of note is that the patient had to be transfused with red cells during her hospitalization. Because of the history of macrocytosis, a BM biopsy was conducted during her hospitalization and MDS with multilineage dysplasia was diagnosed. More specifically, the BM showed a hypercellular picture because of hyperplasia of all three hemopoietic cell lineages (Figure 3A). As shown in the characteristic pictures in Figure 3, there was abundance of diversity in the nuclear morphology of the megakaryocytes (multi- or monolobated forms, irregular chromatin distribution), the aggregates of immature myeloid precursors in the intertrabecular region after myeloperoxidase stain, and the abnormal localization of erythroblasts (some with megaloblastic features) on the endosteum of the trabecular bone after Glycophorin C stain. The immunophenotype showed 0.4% blasts in the BM; thus, the Revised International Prognostic Scoring System (IPSS-R) (6) category of the patient, including her age, was “low.”

**Figure 4:**
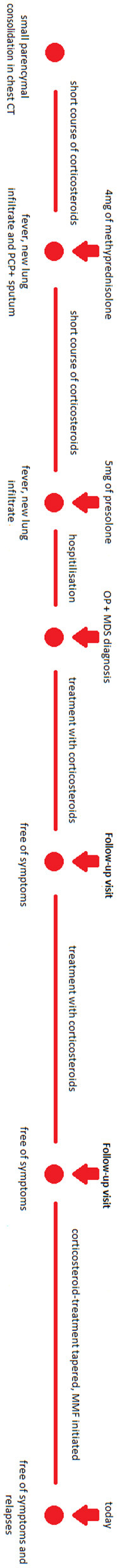
Timeline with relevant data from the episode of care.

The patient was discharged, no longer needing oxygen therapy, with a trimethoprim/sulfamethoxazole prophylactic regimen, prednisolone for OP, and erythropoietin, gastroprotection, and prophylaxis for osteoporosis. Almost 20 days following her discharge in a follow-up visit, the patient had pulmonary function tests with forced vital capacity (FVC) of 103%, forced expiratory volume in 1 s (FEV1) of 106%, an FEV1/FVC ratio of 82%, and total lung capacity (TLco) of 99%. Her ABGs at room air revealed PO_2_ of 81 mmHg, PCO_2_ of 32 mmHg, (pH 7.49), and HCO_3_ of 23 mmol/L. A CT-guided biopsy (Figure 1D) was conducted, which was suggestive of OP with mild chronic interstitial inflammatory infiltration and foci of foamy macrophages. Cortisone therapy was gradually declined and replaced, because of the age of the patient, osteoporosis, and cataract, with MMF without adverse events or relapses. Since her discharge and until today, the patient is regularly monitored, has not re-experienced fever, respiratory failure, or new infiltrate in chest imaging, has low blood cell counts, and is in treatment with erythropoietin. Figure 4 summarizes the timeline of this case report.

## Discussion

This interesting case demonstrates that a new lung infiltrate may be the first manifestation, in the context of the immunological deregulation of myelodysplasia, of a patient who has not been diagnosed and not treated for MDS before. Thus, the clinician must contemplate on their differential diagnosis of AFOP secondary to myelodysplasia when having a patient presenting with respiratory failure and consolidation refractory to antibiotics. The good outcome compared to that in the literature points to the importance of the early initiation of corticosteroid treatment, while the successful switch to MMF indicates the usefulness of this agent in these patients.

As described before in a series of patients with MDS by Enright et al. (7), Enright and Miller (8), and Saif et al. (9), immune manifestations are quite common in patients with myelodysplasia, they are not correlated with the type of MDS, are usually related to worse outcomes, and can be stratified into five categories, i.e., acute systemic vasculitis or autoimmune disorder, chronic or isolated autoimmune phenomena, classical connective tissue disorders, immune-mediated hematological abnormalities, and asymptomatic serological immunological abnormalities. Especially, acute systemic vasculitis, presented as Sweet's syndrome, may include lung infiltrates, as described by Garg et al. (11), Nishimoto et al. (12), and Tzelepis et al. (10).

Matsushima et al. have also described three patients who had a history of refractory anemia and who presented with fever and pulmonary disorders that responded to steroids; as explained, this immunologic abnormality was, in all cases, not isolated, as in our case, but accompanied by systemic vasculitis (13). These patients presented with skin abnormalities, i.e., Sweet's syndrome, bone marrow eosinophilia, polyclonal hypergammaglobulinemia, and elevated plasma cells in the bone marrow. Similarly, the patient of Merrill et al. who was diagnosed with MDS presented with a dominant autoimmune phenotype and with several autoimmune phenomena besides the pulmonary involvement, i.e., symptomatic anemia, lacrimal gland pseudotumor, and thickening of the temporal arteries (15). However, isolated lung infiltrates without other immune manifestations, as in our case, in a newly diagnosed MDS patient have not yet been described in the literature.

Common in the literature is the appearance of lung infiltrates and fever because of infection in those immunodeficient patients, as what Wohlrab et al. described for a patient with pulmonary mucormycosis (16), or the appearance of OP related to an agent given for MDS treatment, as what Vasu et al. described for a patient who received decitabine (17). On the contrary, our patient had negative microbiological testing and had never received an agent related to such adverse effects.

Patients already diagnosed with MDS may present years or months after diagnosis with pulmonary infiltrates that are refractory to antibiotics and other causes are excluded, as described by Shimanuki et al. (18) and Yamakawa et al. (19), unlike our patient who had not yet been diagnosed. Yamamoto et al. had described a patient already diagnosed years before MDS presenting with fever, lung infiltrates, and lymphocytopenia with a histological pattern typical of AFOP. Although the patient initially responded well to corticosteroids and was discharged from the hospital, he died 5 months later (14), unlike our patient who is still in good condition 1.5 years after the episode.

The good outcome of our patient can be ascribed firstly to the early initiation of steroids, in contrast to the relapse described in one MDS patient of Kobara et al. who presented with pulmonary disease and received no steroid treatment (20), and to the possibility that our patient suffered from subacute rather than acute AFOP, which is related to higher mortality rates. Moreover, the case of Kamiya et al. whose MDS patient developed cryptococcosis during corticosteroid treatment for OP (21), strengthens the value of our observation that patients with MDS-related AFOP or OP can safely be switched from corticosteroids to MMF, so avoiding the adverse outcomes of steroids such as infections, osteoporosis, and cataract.

Although this case disposes the strengths analyzed above, a useful add-on would have been if we had obtained a bronchoalveolar lavage (BAL) of the patient. Unfortunately, as usually occurs in clinical practice, the respiratory failure of the patient made bronchoscopy not possible. However, as explained before, BAL is rarely diagnostic because of the patchy histological pattern of AFOP (1). This is also a reason why CT-biopsy can be non-diagnostic as well. Moreover, the fact that FNB was conducted more than 20 days after the initiation of corticosteroids explains why the pathologist described a classical pattern of OP, as this is the healing course of AFOP. Finally, FNB was beneficial over bronchoscopy in our case as it ruled out the possibility of an underlying malignancy in our patient since no such histological findings were present.

In the limitations of the study, it should be noted that, unfortunately, although the examination was requested, karyotype was not completed because of technical reasons. However, in this case, the macrocytosis of the patient (elevated MCV in the PB with normal values of hemoglobin and hematocrit for the last years) preceded any infection or any manifestation of the lung, and thus, the possibility of infection causing dysplastic abnormalities of the BM seems unlikely.

In conclusion, this case report suggests firstly that a diagnosis of AFOP or OP should alert the clinician to search for an underlying cause including MDS and *vice versa*, that the use of systemic steroids should not be postponed, and, finally, that MMF can successfully be used in these patients.

## Data Availability Statement

The original contributions presented in the study are included in the article/supplementary material, further inquiries can be directed to the corresponding author/s.

## Ethics Statement

Written informed consent was obtained from the individual(s) for the publication of any potentially identifiable images or data included in this article.

## Author Contributions

NB, GP, DI, GS, EV, SH, MB, KK, MF, KA, and NT observed and treated the patient. KL and IX conducted the BM biopsy and did hematological follow-up of the patient. LM is the pathologist who made the diagnosis of MDS and added the pictures of the biopsy in the manuscript. NB and GP reviewed the literature and wrote the paper. KA and NT supervised the work. All authors approved the final work.

## Conflict of Interest

The authors declare that the research was conducted in the absence of any commercial or financial relationships that could be construed as a potential conflict of interest.

## Publisher's Note

All claims expressed in this article are solely those of the authors and do not necessarily represent those of their affiliated organizations, or those of the publisher, the editors and the reviewers. Any product that may be evaluated in this article, or claim that may be made by its manufacturer, is not guaranteed or endorsed by the publisher.
